# Effect of systemic therapies or best supportive care after disease progression to both nivolumab and cabozantinib in metastatic renal cell carcinoma: The Meet‐Uro 19BEYOND study

**DOI:** 10.1002/cam4.4681

**Published:** 2022-03-20

**Authors:** Giandomenico Roviello, Elisabetta Gambale, Roberta Giorgione, Daniele Santini, Marco Stellato, Giuseppe Fornarini, Sara Elena Rebuzzi, Umberto Basso, Davide Bimbatti, Laura Doni, Gabriella Nesi, Melissa Bersanelli, Sebastiano Buti, Ugo De Giorgi, Luca Galli, Andrea Sbrana, Raffaele Conca, Claudia Carella, Emanuele Naglieri, Sandro Pignata, Giuseppe Procopio, Lorenzo Antonuzzo

**Affiliations:** ^1^ Department of Health Sciences University of Florence Florence Italy; ^2^ Medical Oncology Unit Careggi University Hospital Florence Italy; ^3^ Department of Medical Oncology University Campus Bio‐Medico Rome Italy; ^4^ Medical Oncology Unit 1 IRCCS Ospedale Policlinico San Martino di Genova Genoa Italy; ^5^ Medical Oncology Unit San Paolo General Hospital Savona Italy; ^6^ Department of Internal Medicine and Medical Specialties (Di.M.I.) University of Genova Genoa Italy; ^7^ MedicalOncology Unit 1 Istituto Oncologico Veneto IOV IRCCS Padua Italy; ^8^ Medical Oncology Unit University Hospital of Parma Parma Italy; ^9^ IRCCS Istituto Romagnolo per lo Studio dei Tumori (IRST) "Dino Amadori" Italy; ^10^ Medical Oncology Unit 2 Azienda Ospedaliero‐Universitaria Pisana Pisa Italy; ^11^ Department of Surgical, Medical and Molecular Pathology and Critical Care Medicine University of Pisa Pisa Italy; ^12^ Division of Medical Oncology, Department of Onco‐Hematology IRCCS‐CROB, Referral Cancer Center of Basilicata Rionero in Vulture Italy; ^13^ Istituto Oncologico Giovanni Paolo II Bari Italy; ^14^ Department of Urology and Gynecology Istituto Nazionale Tumori IRCCS Fondazione G. Pascale Naples Italy; ^15^ Department of Medical Oncology Fondazione IRCCS Istituto Nazionale dei Tumori di Milano Milan Italy; ^16^ Department of Experimental and Clinical Medicine University of Florence Florence Italy

**Keywords:** fourth‐line therapy, immune checkpoint inhibitors, metastatic renal cell carcinoma, targeted therapy, tyrosine kinase inhibitors, vascular endothelial growth factor receptor

## Abstract

**Background:**

Nivolumab and cabozantinib are currently approved agents in metastatic renal cell carcinoma (mRCC) but there are no data available for patients progressing to both treatments. The aim of this study was to compare active therapeutic options and best supportive care (BSC) after progression to nivolumab and cabozantinib in mRCC.

**Methods:**

In this retrospective study, we selected 50 patients from eight Italian centers. The primary endpoint of the study was the overall survival (OS) of patients on active treatment versus BSC. Secondary endpoints were the progression‐free survival (PFS) and objective response rate (ORR). The efficacy of active therapy was also investigated.

**Results:**

After progression to both nivolumab and cabozantinib, 57.1% of patients were given active treatment (mainly everolimus and sorafenib) while 42.9% received BSC. The median OS was 13 months (95% CI: 4‐NR) in actively treated patients and 3 months (95% CI: 2–4) in BSC patients (*p* = 0.001). Patients treated with sorafenib had better disease control than those treated with everolimus (stable disease: 71.4% vs. 16.7%, progression disease: 14.3% vs. 58.3%; *p* = 0.03), with no significant differences in PFS (5 and 3 months, 95% CI: 1–6 vs. 2–5; *p* = 0.6) and OS (12 and 4 months, 95% CI: 3‐NR vs. 2‐NR; *p* = 0.2).

**Conclusion:**

After treatment with both nivolumab and cabozantinib, the choice of a safe active systemic therapy offered better outcomes than BSC.

## Graphical abstract

In this paper, we report the results of our retrospective analysis of mRCC treated before the approval of 1 L‐ anti‐PD1 based combination, with the aim to compare the treatment choices after progression to nivolumab and cabozantinib including patients followed in BSC or active therapy.

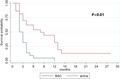

## 1 INTRODUCTION

In 2020, approximately 431,000 new cases of kidney cancer were diagnosed worldwide, resulting in 179,000 deaths.^1^ Renal cell carcinoma (RCC) is the most common type, accounting for almost 90% of kidney cancers, and the clear cell RCC (ccRCC) is the most frequent histology (70%–90%).^2^ The Memorial Sloan Kettering Cancer Center (MSKCC) and the International Metastatic Renal Cell Carcinoma Database Consortium (IMDC) risk models are being used to predict the outcome of mRCC patients treated with systemic therapies.

Recently, two‐phase III large trials compared nivolumab (antibody against programmed death 1 receptor, PD‐1) or cabozantinib (multityrosine kinase inhibitor, TKI) versus everolimus, showing significant improvement in overall survival (OS) and objective response rate (ORR). Cabozantinib also improved progression‐free survival (PFS) in comparison to everolimus. In both trials, patients may have previously received one or two TKIs.^3^, ^4^, ^5^

Updates on the combination of anti‐PD‐1 and anti‐vascular endothelial growth factor receptor (VEGFR) have led to the design of new algorithms of care for mRCC.^6^, ^7^ In particular, pembrolizumab together with axitinib (PA) is now one of the recommended front‐line/treatment‐naïve therapies for mRCC according to the results of KEYNOTE‐426. In this phase III trial, PA was compared with sunitinib in previously untreated mRCC patients.^8^ Benefits of this combination appeared to be independent of IMDC prognostic subgroups and programmed death‐ligand 1 (PD‐L1) expression.^8^ Another combination, nivolumab and cabozantinib (NC), is currently recommended as front‐line therapy for mRCC based on results from the CheckMate 9ER study.^7^ This trial showed superiority of NC over sunitinib in the first‐line (1 L) setting regardless of PD‐L1 expression, and efficacy benefits were consistent across IMDC subgroups.^9^

Randomized trials of axitinib plus avelumab (anti‐PD‐L1) and bevacizumab plus atezolizumab (anti‐PD‐L1) have also been conducted in the 1 L setting.^10^, ^11^ These combinations were tested against sunitinib achieving their pre‐defined PFS co‐primary endpoint, although no statistical significance in OS was recorded. Some exploratory analysis with extended follow‐up confirmed long‐term efficacy and safety, but final OS data are still awaited.^11^, ^12^ A further immunotherapy (IO)/TKI combination was evaluated in the CLEAR study, where patients with untreated advanced RCC were enrolled to receive lenvatinib plus pembrolizumab or lenvatinib plus everolimus, or else sunitinib alone. The IO/TKI combination was associated with significantly longer PFS (primary endpoint) and OS than sunitinib.^13^

Moreover, the anti‐cytotoxic T‐lymphocyte antigen 4 (CTLA‐4) ipilimumab combined with nivolumab (IN) was investigated in the phase III CheckMate214 trial.^14^, ^15^ At a 4‐year median follow‐up, only the IMDC intermediate‐ and poor‐risk groups benefited from the IN regimen.^7^, ^8^, ^12^

Despite these encouraging results, many patients require subsequent therapies after discontinuation of front‐line immune checkpoint inhibitor (ICI) based combinations.^16^ Therefore, additional data to clarify the optimal strategy are mandatory, and the most appropriate treatment patterns in fourth line (4 L) and beyond settings need to be investigated.

While the European Society of Medical Oncology (ESMO) has provided guidelines for the choice of treatment in the first, second, and third lines,^6^, ^7^ there is no solid evidence on how to select the best treatment beyond the third line. Although RECORD‐1, METEOR, and TIVO‐3 studies involved patients receiving everolimus or cabozantinib as fourth‐line (4 L) therapy, results were ill defined owing to the small number of patients enrolled.^4^, ^5^, ^17^, ^18^

In this complex scenario, we report the findings of our retrospective analysis of mRCC patients treated before the approval of 1 L‐ anti‐PD1 based combination, aiming to compare active treatments to BSC after disease progression to nivolumab and cabozantinib.

## 2 MATERIALS AND METHODS

### 2.1 Study population

We retrospectively collected data of mRCC patients progressed to both nivolumab and cabozantinib, and subsequently undergoing active treatment or BSC in eight Italian referral centers adhering to the Meet‐Uro group, between October 2017 and January 2020. Inclusion criteria were as follows: age ≥ 18 years old, histologically confirmed diagnosis of RCC, evidence of metastatic disease, and history of disease progression after previous lines of treatment with nivolumab and cabozantinib. Baseline data were collected after initial mRCC diagnosis and prior to systemic therapy initiation.

Patients were stratified into three risk groups (favorable, intermediate, and poor) according to the MSKCC prognostic model^19^ proposed and validated before the advent of targeted agents. This model is based on five independent risk factors: low Karnofsky Performance Status (KPS, <80%), low serum hemoglobin (less than the lower limit of normal), high lactate dehydrogenase (LDH, >1.5× upper limit of normal), high corrected serum calcium (>10 mg/dl), and time from diagnosis to systemic treatment less than 1 year. The favorable‐risk group includes patients with no risk factors, the intermediate‐risk group comprises patients with one or two risk factors, while the poor‐risk group includes patients with three and more risk factors.

### 2.2 Assessment and clinical outcomes

Tumor response evaluation was generally performed by spiral chest and abdomen computed tomography approximately every 3 months, or before if progression was suspected, in line with local clinical practice. Disease progression was assessed either radiologically or clinically, if imaging was not available due to rapid clinical progression of the disease. The best response during active treatment was radiologically estimated according to the Response Evaluation Criteria in Solid Tumors (RECIST) version 1.1.^20^ The primary endpoint of the study was OS of patients on active treatment and BSC. Secondary endpoints were tumor response, PFS, and OS of patients on active treatment, who were administered sorafenib and everolimus.

OS was defined as time from active treatment or BSC initiation to death from any cause or censored at the time of last follow‐up. PFS was defined as the time from active treatment or BSC initiation until date of first evidence of disease progression or death from any cause. Tumor response was defined as the proportion of patients with the best overall response determined as complete response (CR), partial response (PR), or stable disease (SD).^20^ ORR and progressive disease (PD) were assessed per RECIST version 1.1.^20^ For patients treated with nivolumab, immune (i)‐RECIST was used to confirm PD.^21^ The Kaplan–Meier method was performed to estimate OS and PFS. The median of each endpoint was calculated with their 95% confidence intervals (CI). For other parameters, quantitative variables were summarized using descriptive statistics, and categorical variables were summarized using numbers and percentages. A two‐sided *p* < 0.05 was considered statistically significant. All statistical analyses were carried out using STATA, version 12.0; StataCorp. This study was conducted in compliance with the ethical standards recommended by the Declaration of Helsinki, and was approved by the Comitato Etico Regionale for clinical experimentation of the Tuscany Region (Italy) Area Vasta Centro Section**, number:16813_oss**. The decision to participate in this study was voluntary, and written informed consent required by the Ethics Committees was obtained from all participants.

## 3 RESULTS

Data from 50 mRCC patients were retrospectively collected from eight referral centers. The median age was 65 years and 78% of patients were male. The majority of patients (86%) were classified as intermediate and poor risk at diagnosis. Overall, 78% of patients had undergone nephrectomy and 82% had clear cell histology. The lungs were the most frequent site of metastatic disease in the general population (72%) and in patients referred to BSC (86.4%), while bone metastases were prevalent in actively treated patients (67.9%). Sunitinib (66%), nivolumab (60%), and cabozantinib (52%) were the most widely used agents in the first‐, second‐, and third‐line settings, respectively.

Patients progressing to both nivolumab and cabozantinib received BSC (44%) or active treatment (56%), including everolimus (26%), sorafenib (14%), sunitinib (8%), high‐dose interleukin‐2 (IL‐2) (4%), or lenvatinib plus everolimus (2%). In the BSC group, five patients received cabozantinib as 4 L therapy and one patient received cabozantinib in sixth line (6 L) while in the active group, seven patients received cabozantinib in 4 L. In the BSC group, one patient received nivolumab as 4 L therapy, and in the active group, no patients were administered nivolumab as 4 L or subsequent systemic therapy. Finally, almost all patients in the BSC group had an ECOG PS of 2 (95.5%) versus 14.3% of patients who were given active treatment. Patient characteristics are detailed in Table 1.

**TABLE 1:** Patients' baseline characteristics

	All patients (50)	Active (28)	BSC (22)	*p* value
Age				
Median	65	63.5	65	0.7
Range	43–85	44–79	43–85	
Gender				
Male	39 (78%)	23 (82.1%)	16 (72.7%)	0.4
Histology				
Clear‐cell RCC	41 (82%)	23 (82.1%)	18 (81.8%)	0.9
Previous surgery				
Yes	39 (78%)	22 (78.6%)	17 (77.3%)	0.6
Site of metastasis				
Lung	36 (72%)	17 (60.7%)	19 (86.4%)	0.03
Bone	30 (60%)	19 (67.9%)	11 (50%)	0.1
Lymph nodes	34 (68%)	16 (57.1%)	18 (81.8%)	0.1
Liver	27 (54%)	12 (42.9%)	15 (68.2%)	0.1
Other	34 (68%)	16 (57.1%)	18 (81.8%)	0.1
ECOG				
0	8 (16%)	8 (28.6%)	0	<0.01
1	17 (34%)	16 (57.1%)	1 (4.5%)	
>2	25 (50%)	4 (14.3%)	21 (95.5%)	
MSKCC score				
Good	7 (14%)	5 (17.9%)	2 (9.1%)	0.3
Intermediate‐poor	43 (86%)	23 (82.1%)	20 (90.9%)	
First‐Line Therapy				
Sunitinib	33 (66%)	16 (57.1%)	17 (77.3%)	0.3
Pazopanib	13 (26%)	10 (35.7%)	3 (13.6%)	
Other	4 (8%)	2 (7.2%)	2 (9.1%)	
Second‐line				
Cabozantinib	11 (22%)	6 (21.4%)	5 (22.7%)	0.9
Nivolumab	30 (60%)	18 (64.3%)	12 (54.5%)	
Other	9 (18%)	4 (14.3%)	5 (22.8%)	
Third line				
Cabozantinib	26 (52%)	15 (53.6%)	11 (50%)	0.9
Nivolumab	19 (38%)	10 (35.7%)	9 (40.9%)	
Other	5 (10%)	3 (10.7%)	2 (9.1%)	
Line of treatment after cabozantinib and nivolumab				
4	37 (74%)	21 (75%)	16 (72.7%)	0.6
5	11 (22%)	7 (25%)	4 (18.2%)	
>5	2 (4%)	0	2 (9.1%)	
Treatment after cabozantinib and nivolumab BSC 22 44% IL‐2 HD 2 4% everolimus 13 26% lenvatinib+eve 1 2% sorafenib 7 14% sunitinib 4 8% other 1 2%				

Abbreviations: BSC, Best Supportive Care; ECOG PS, Eastern Cooperative Oncology Group Performance Status; IL2‐HD, High Dose Interleukin 2; MSKCC score, Memorial Sloan–Kettering Cancer Center; RCC, Renal Cell Carcinoma PD, progressive disease.

**FIGURE 1:**
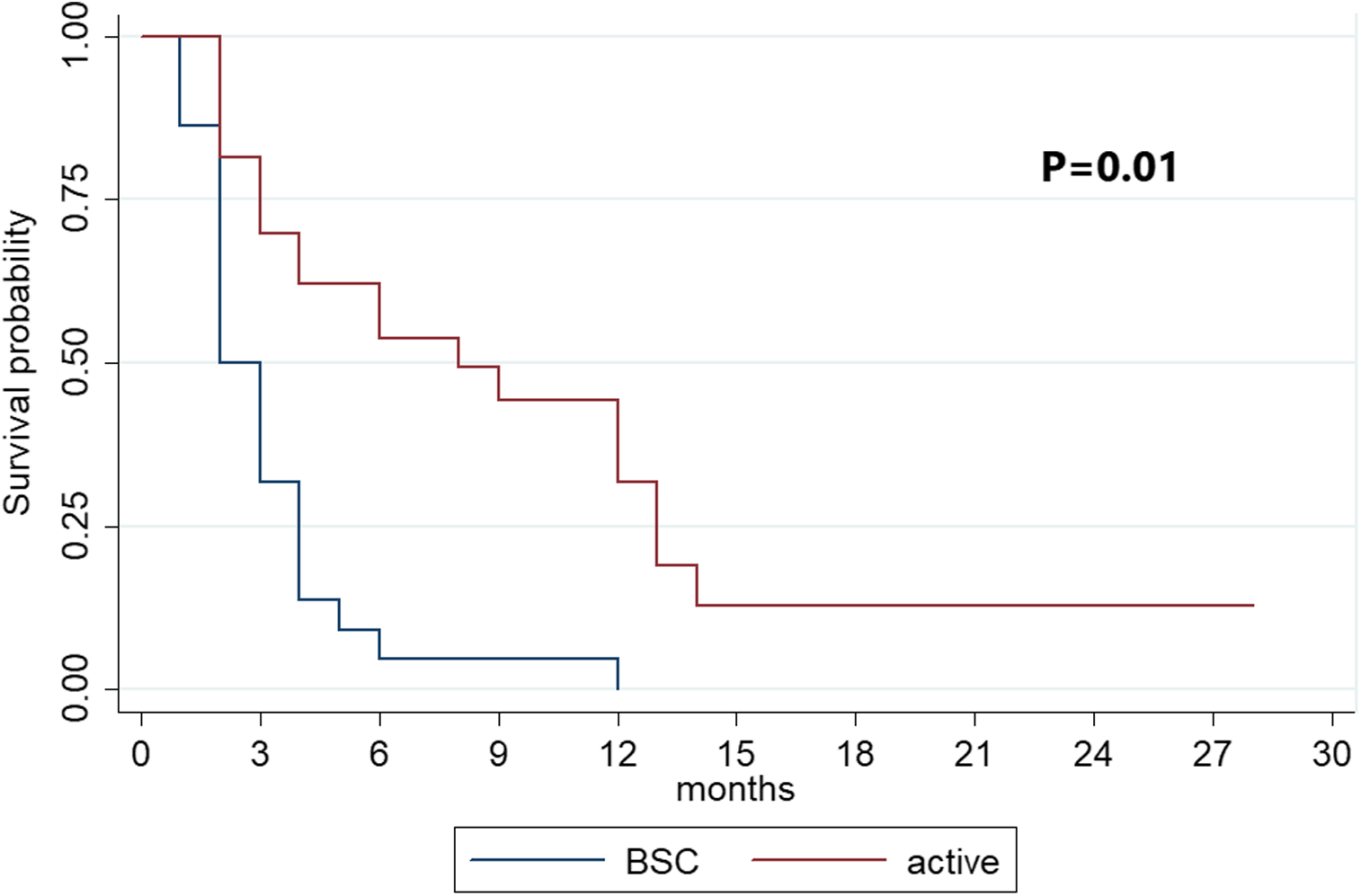
Overall Survival (OS) of patients on Best Supportive Care (BSC) versus patient on active treatment

As outlined in Table 2 and Figure 1, the median OS was 8 (95% CI: 3–13) and 2 months (95% CI: 2–4) in patients undergoing active treatment and BSC, respectively (*p* < 0.001). Regarding efficacy outcomes of subsequent treatments, patients treated with sorafenib showed better disease control than those treated with everolimus (SD 83.3% and 20%, PD 16.7% and 80%; *p* = 0.02). No significant difference in PFS (5 and 3 months, 95% CI: 1–6 vs. 2–5; *p* = 0.6) and OS (12 and 4 months, 95% CI: 3‐NR vs. 2‐NR; *p* = 0.2) was observed (Table 3 **)**,

**TABLE 2:** Survival of the study population

	All patients (*n* = 50)	Active treatment (*n* = 28)	BSC (*n* = 22)	*p*
mOS‐months	4	8	2	<0.001
95% CI	3–6	3–13	2–4	
SEQ Nivolumab‐cabozantinib	4	8	2	<0.001
95% CI	3–6	3–13	2–4	
SEQ Cabozantinib‐nivolumab	3	9	2	
95% CI	2–9	2‐ NR	1‐NR	

Abbreviations: BSC, best supportive care; CI, confidence interval; mOS, median overall survival; *n*, number of included patients; NR, not reached; SEQ, sequential.

**TABLE 3:** Best response (according to RECIST 1.1 criteria) in the population of patients on active treatment; progression‐free survival (PFS) and overall survival (OS) results according to sorafenib and everolimus

	Sorafenib (*n* = 7)	Everolimus (*n* = 13)	*p*
PR	0	0	0.02
SD			
*n* (%)	5 (83.3)	2 (20)	
PD			
*n* (%)	1 (16.7)	8 (80)	
NE	1 (14.3)	3 (23.1)	
mPFS			0.6
Months	5	3	
(95% CI)	(1–6)	(2–5)	
mOS			0.2
Months	12	4	
95% CI	3‐NR	2‐NR	

Abbreviations: CI, confidence interval; mOS, median overall survival; mPFS, median progression‐free survival; *n*, number of included patients; PD, progression disease; PR, partial response; SD, stable disease.

## 4 DISCUSSION

A systemic treatment beyond the 3 L is currently offered to a large number of mRCC patients, nevertheless the optimal therapy for the fourth and subsequent lines remains unknown. This is partially due to the exclusion of heavily pre‐treated patients from most clinical trials, which are predominantly arranged in the 1 and 2 L settings. The 4 L setting has not been extensively investigated and these data are mostly from retrospective studies, which do not include patients who progressed to both nivolumab and cabozantinib.^4^, ^5^, ^16^, ^17^, ^22^, ^23^, ^24^, ^25^

In our study, survival outcomes were in line with those reported by IMDC for a largely heterogeneous patient population (*n* = 594; OS = 12.8 months) receiving different targeted agents in the 4 L treatment.^26^ Similar survival data were recorded by Ralla et al. in a retrospective German study, with a median OS of 10.5 months in patients administered a 4 L therapy (*n* = 56) and 6.2 months for those on a fifth‐line (5 L) therapy (*n* = 25) (IQR 3.1–23.8).^27^, ^28^ Notably, our analysis included more than 25% of patients treated beyond the 4 L setting. Recently, Cerbone et al. conducted a retrospective study on heavily pre‐treated mRCC patients undergoing systemic therapy after cabozantinib failure. These authors found an OS of 7.7 months and an ORR of 8.7%, achieving PR in two patients on axitinib and two on ICIs.^29^

In agreement with previous reports, our findings show that everolimus is the most common 4 L and beyond treatment, followed by sorafenib.^26^ Everolimus was investigated in the phase III double‐blind, randomized, placebo‐controlled RECORD‐1 in patients who progressed under treatment with one or two TKIs, leading to improved PFS (4.0 months vs. 1.9 months; HR, 0.33; *p* < 0.001).^30^ Based on this evidence, everolimus received approval as a potential systemic treatment in refractory settings.^30^ In clinical practice, everolimus is only given to highly therapy‐refractory patients, due to the development of more active agents in mRCC.^23^ Regarding the use of everolimus post ICI progression, there are currently no prospective data. Retrospective studies suggest its efficacy in patients requiring additional therapy after PD‐1/PD‐L1 blockade, although 1‐year OS is poorer (27%) than with axitinib (67%) or sorafenib (61%).^31^, ^32^ Nivolumab and cabozantinib are recommended in the 3 L setting,^7^ and available information on sorafenib as 4 L therapy does not cover any previous disease progression to both nivolumab and cabozantinib.^17^ Since there is no clear evidence to suggest superiority of any treatment in the 4 L setting, choice will continue to be based on the drugs available and the best OS achieved with TKIs. Identification of those patients most likely to benefit from 4 L and further treatments is critical to ensure the best possible outcomes. As shown in Table 1, PS appears to guide clinical management, in fact, most patients in the BSC group have PS = 2. Based on our data, we suggest treating only patients with PS < 2 in the 4 L and 5 L settings. The discovery and use of novel biomarkers for therapy selection will, hopefully, allow determining the ideal treatment patterns in the 1 L, 2 L, 3 L, 4 L and beyond settings. Of note, in our study, a considerable number of patients at intermediate/poor risk (82.1%) received four or more systemic therapies.

Our study has several limitations, including treatment selection bias, lack of data on novel combinatorial regimens, small sample size, and its retrospective design. Nonetheless, to the best of our knowledge, this is the first retrospective analysis to include patients pre‐treated with sequential novel agents, such as cabozantinib and nivolumab, providing further evidence on the benefits of 4 L and beyond systemic therapies.

Prospective data and study design to explore this therapeutic setting are compulsory. As of today, an increasing number of clinical trials, summarized in Table 4, have recruited patients in the 4 L and beyond setting. It will be of interest to evaluate the clinical results from the different therapeutic sequences used, and which of these lead to survival improvements. However, the considerable variability in tumor biology across patients and tumor types calls for the recognition of biomarkers to warrant patient selection.

**TABLE 4:** Selected 4 L and beyond treatment trials formRCC^a^

NCT Trial	Trial	Phase	Status
NCT02926053	T Cell Therapy for Patients With Metastatic Renal Cell Carcinoma	I	Recruiting
NCT03967522	Evaluation of Cabozantinib in Metastatic Renal Cell Carcinoma (mRCC) With Brain Metastases (CABRAMET)	II	Recruiting
NCT04262427	Cyclophosphamide And PEmbrolizumab in Metastatic Renal Cell Carcinoma (CAPER)	Ib	Not yet recruiting
NCT04068831	Talazoparib and Avelumab in Participants With Metastatic Renal Cell Carcinoma	II	Recruiting
NCT04049344	Decitabine Combined With Oxaliplatin in Patients With Advanced Renal Cell Carcinoma	II	Recruiting
NCT03786796	Study of Olaparib in Metastatic Renal Cell Carcinoma Patients With DNA Repair Gene Mutations (ORCHID)	II	Recruiting
NCT03138538	Dose Escalation Trial of Methionine Aminopeptidase 2 Inhibitor M8891 in Subjects With Advanced Solid Tumors	I	Active not recruiting
NCT03987698	Clinical Study of anti‐PD‐1 Monoclonal Antibody Combination camrelizumab With Autologous Cytokine‐induced Killer Cell Immunotherapy in the Second‐line Treatment of Metastatic Clear Cell Renal Cell Carcinoma	II	Recruiting
NCT03071328	Pilot Study of Intra‐tumoral Injections of Isovue‐M 200in Metastatic Urological Cancers	I	Recruiting
NCT04603365	Pamiparib and Temozolomide for the Treatment of Hereditary Leiomyomatosis and Renal Cell Cancer	II	Not yet recruiting
NCT03682289	Trial of AZD6738 Alone and in Combination With Olaparib	II	Recruiting
NCT04140526	Safety, PK and Efficacy of ONC‐392 (anti‐CTLA4 mAb) in Monotherapy and in Combination of Anti‐PD‐1 in Advanced Solid Tumors and NSCLC (PRESERVE‐001)	I	Recruiting
NCT03294083	A Study of Recombinant Vaccinia Virus in Combination WithCemiplimab for Renal Cell Carcinoma	1b/2a	Recruiting
NCT04628780	Study to Test the Safety and Tolerability of PF‐07209960 in Advanced or Metastatic Solid TumorsPF‐07209960, an anti‐PD‐1 targeting IL‐15 fusion protein	I	Recruiting
NCT04198766	Study of INBRX‐106 and INBRX‐106 in Combination With Pembrolizumab in Subjects With Locally Advanced or Metastatic Solid Tumors (Hexavalent OX40 Agonist)	I	
NCT04152018	Study of PF‐06940434 (antagonist of integrin alpha v beta 8) in Patients With Advanced or Metastatic Solid Tumors.	I	Recruiting

^a^ as of Aug 11, 2021. Source: clinicaltrials.gov.

## 5 CONCLUSIONS

Compared with BSC, active systemic therapy appears to offer an OS advantage in patients receiving nivolumab and cabozantinib. Both sorafenib and everolimus are possible treatment options, although in these patients it cannot be determined which one is superior. Further studies are required to identify the optimal drug combination, appropriate timing of administration, and best therapeutic sequence in mRCC patients. In the meantime, ongoing research will shed light on the mRCC treatment paradigm and help elucidate the preferred agent.

## CONFLICT OF INTEREST

No author declares any conflict of interest.

## AUTHOR CONTRIBUTIONS

Giandomenico Roviello had full access to all the data in the study and takes responsibility for the integrity of the data and the accuracy of the data analysis. Study concept and design: RG. Acquisition of data: EG, RG. Recruiting patients: DS, MS, GF. SER, UB, DB, LD, GN, MB,SB, UDG, LG, AS, RC, CC, EN, SP, GP. Analysis and interpretation of data: RG. Drafting of the manuscript: RG, EG, RG. Critical revision of the manuscript for important intellectual content: GR, LA. Statistical analysis: RG.

## ETHICS STATEMENT

All procedures performed in studies involving human participants were in accordance with the ethical standards of the institutional and/or national research committee and with the 1964 Helsinki declaration and its later amendments or comparable ethical standards.

## Data Availability

The data used to support the findings of this study are available from the corresponding author upon request.
